# A New Cytotoxic Pregnanone from *Calotropis gigantea*

**DOI:** 10.3390/molecules13123033

**Published:** 2008-12-04

**Authors:** Zhu-Nian Wang, Mao-Yuan Wang, Wen-Li Mei, Zhuang Han, Hao-Fu Dai

**Affiliations:** 1 Institute of Crops Genetic Resources, Chinese Academy of Tropical Agricultural Sciences, Danzhou, 571737, P. R. China; E-mails: wmy81@163.com (M-Y. W.); wangzhunian@yahoo.com.cn (Z-N. W.); 2 Institute of Tropical Bioscience and Biotechnology, Chinese Academy of Tropical Agricultural Sciences, Haikou, 571101, P. R. China; E-mails: hanzone@yahoo.cn (Z. H.); meiwenli@yahoo.com.cn (W-L. M.)

**Keywords:** *Calotropis gigantea* L., Calotropone, Cytotoxicity.

## Abstract

A new pregnanone, named calotropone (**1**), was isolated from the EtOH extract of the roots of *Calotropis gigantea* L. together with a known cardiac glycoside. The structures were elucidated by a study of their physical and spectral data. Compounds **1** and **2** displayed inhibitory effects towards chronic myelogenous leukemia K562 and human gastric cancer SGC-7901 cell lines.

## Introduction

The genus *Calotropis* (Asclepiadaceae) is comprised of about six species of shrubs distributed throughout tropical and subtropical Africa and Asia. Two of them, *Calotropis gigantea* L. and *Calotropis procera* L. occur in China, and are two sister species. *C. gigantea* is a high biomass, fast growing perennial shrub growing as a weed in the Hainan province of China [1]. It was used as a traditional folk medicine for the treatment of anthelmintic, carminative, cough, leprosy, and asthma by the people of the Li nationality, who are autochthonous to Hainan island in China. The chemical constituents of *C*. *gigantea* have been extensively investigated, leading to the isolation of many cardenolides [2,3,4,5], flavonoids [6], terpenes [7,8,9,10], pregnanes [11,12] and a nonprotein amino acid [13]. During our screening for cytotoxic agents from tropical medicinal plants, the ethanol extract of the roots of *C. gigantea* showed cytotoxic activity towards human chronic myelogenous leukemia (K562) and human gastric cancer (SGC-7901) cell lines *in vitro* by MTT method with IC_50_ values of 9.7 *μ*g/mL and 6.7 *μ*g/mL, respectively. Bioassay-guided fractionation led to the isolation of a new pregnanone, calotropone, together with one known cardiac glycoside gofruside (**2**) from the ethanol extract of *C. gigantea*, their structures were elucidated using spectral means especially 1D and 2D NMR spectroscopy. Both compounds **1** and **2** showed significant cytotoxicity against K562 and SGC-7901 cell lines. In this paper, we describe the isolation, structural elucidation, and cytotoxicity of **1** and **2**.

**Figure 1:**
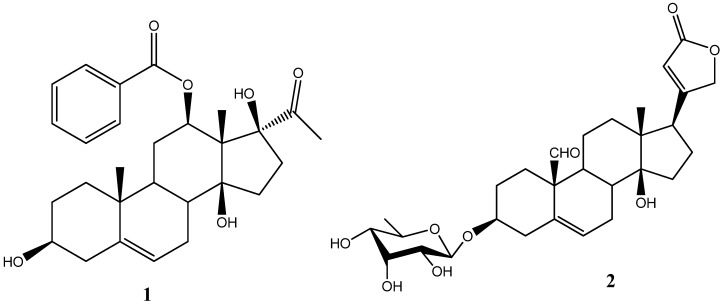
Structures of Compounds **1** and **2**.

## Results and Discussion

**Figure 2:**
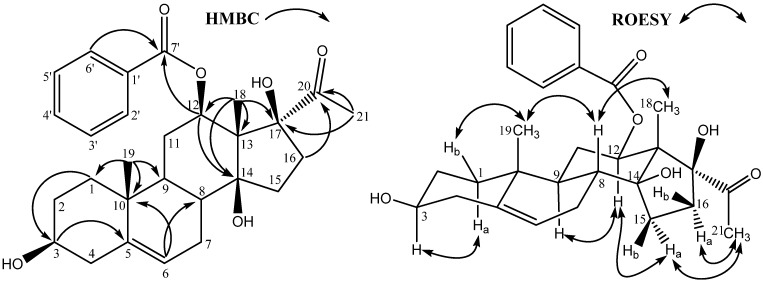
Key HMBC and ROESY correlations of compound **1.**

Bioassay-guided fractionation of the ethanol extract of *C. gigantea* led to the isolation of compounds **1** and **2**. Compound **1** was obtained as yellow gum. The [M+Na]^+^ at *m/z* 491.2409 (calcd 491.2404) in the high-resolution ESI-Mass spectrum corresponded to the molecular formula C_28_H_36_O_6_. This formula can also be validated through ^1^H-NMR, ^13^C-NMR and DEPT spectra. The IR spectrum of **1** showed absorption bands due to a hydroxyl (3,431 cm^-1^) and a carbonyl (1,712 cm^-1^) group, whereas the UV spectrum of **1** suggested the presence of a benzoyl group (absorption maxima at 241, 267, and 284 nm). The ^1^H-NMR spectrum of **1** suggested the presence of a benzoyl group [signals at *δ* 7.43 (2H, t, *J* = 7.5 Hz), 7.56 (1H, t, *J* = 7.5 Hz), 7.93 (2H, d, *J* = 7.5 Hz)]. In addition, one olefinic proton (*δ* 5.41, m) and three high field methyl singlet at δ 2.06, 1.41, and 0.98 were also observed. The ^13^C NMR (DEPT) spectra of **1** showed the coexistence of three methyl groups, seven methylene groups, two aliphatic sp^3^ methine carbons, two oxygenated sp^3^ methine carbons, four sp^3^ quaternary carbon atoms, one tri-substituted double bond, one benzoyl group, and one ketone. This observation suggested that **1** was likely to be a lineolon-type compound. Comparing the ^13^C-NMR spectral data with those of the 12-*O*-benzoyllineolon showed that **1** had one tertiary carbon more and one quarternary carbon less than 12-*O*-benzoyllineolon [12]. The ^1^H-^1^H COSY, HMQC, and HMBC spectra allowed the complete assignments of chemical shifts of **1** (Table 1). The chemical shift of C-8 of **1** was upfield shifted to δ 37.0, which suggested that C-8 was not substituted by a hydroxyl group as 12-*O*-benzoyllineolon. The relative stereochemistry of **1** was determined by ROESY correlations (Figure 2). Based on the above evidence, the structure of compound **1** was identified as 12β-*O*-benzoyl-3β,14β,17β-trihydroxy-pregnane-20-one, named calotropone.

Compounds **1** and **2** were evaluated for their cytotoxic activity against K562 and SGC-7901 cell lines using the MTT method [14], and both of them showed significant cytotoxicity against the two cell lines (Table 2).

**Table 1:** The NMR data of compound **1.**

Position	δ_C_	δ_H_	HMBC
**1**	37.0	1.75 (1H, m, H-1a), 1.13 (1H, m, H-1b)	C-2, 3, 5, 10
**2**	31.4	1.81, 1.46 (each 1H, m)	C-3, 4, 10
**3**	71.4	3.53 (1H, m)	C-1, 2, 5
**4**	41.9	2.33 (1H, dd, 12.8, 3.6 Hz), 2.25 (1H, m, overlapped)	C-2, 6, 5, 10
**5**	139.5		
**6**	121.1	5.41 (1H, m)	C-4, 5, 7, 10
**7**	26.0	2.20, 1.91 (each 1H, m)	C-5, 6, 9, 14
**8**	37.0	1.80 (1H, m)	C-7, 10, 14
**9**	42.6	1.32 (1H, m)	C-1, 5, 11, 12, 14
**10**	36.7		
**11**	26.5	2.06, 1.45 (each 1H, m, overlapped)	C-8, 10, 13
**12**	73.1	4.80 (1H, dd, 11.3, 4.5 Hz)	C-9, 14, 17, 19, 7'
**13**	57.5		
**14**	88.5		
**15**	31.7	2.12 (1H, m, H-15a), 1.92 (1H, m, H-15b)	C-8, 13, 17
**16**	31.8	2.90 (1H, m, H-16a), 1.88 (1H, m, H-16b)	C-13, 14, 20
**17**	91.2		
**18**	7.7	1.41 (3H, s)	C-12, 13, 14, 17
**19**	19.4	0.98 (3H, s)	C-1, 5, 9, 10
**20**	209.3		
**21**	27.4	2.06 (3H, overlapped)	C-17, 20
**1'**	129.9		
**2'**	128.4	7.93 (1H, d, 7.5 Hz)	C-1', 3', 4', 6'
**3'**	129.5	7.43 (1H, t, 7.5 Hz)	C-1', 2', 4', 5'
**4'**	133.2	7.56 (1H, t, 7.5 Hz)	C-2', 3', 5', 6'
**5'**	129.5	7.43 (1H, t, 7.5 Hz)	C-1', 3', 4', 6'
**6'**	128.4	7.93 (1H, d, 7.5 Hz)	C-1', 2', 4', 5', 7'
**7'**	165.3		

The data were measured in CDCl_3_ with reference to TMS.

**Table 2:** IC_50_ values for inhibition of human cell lines of compounds **1** and **2.**

	Compounds (IC_50,_ *μ*g/mL)		
Cell lines	1	2	Mitomycin C^*^
K562	9.2	4.7	7.1
SGC-7901	91.3	14.1	8.8

^*^Mitomycin C (MMC) was used as a positive control.

## Conclusions

Although *C. gigantea* was used as a very famous traditional folk medicine by many cultures, and it has been the subject of extensive phytochemical and bioactive investigations, its chemical components and bioactivities have not been completely investigated yet. Up to now, seven oxypregnane-oligoglycosides, calotroposides A−G have been isolated from the roots of *C. gigantea* [5,11]. In our present study a new pregnanone was isolated and identified from the genus of *Calotropis*, this is the first steroidal aglycone isolated from this genus. Meanwhile, the cytotoxicity against K562 and SGC-7901 cell lines of compounds **1** and **2** was evaluated for the first time, this is also the first report about the cytotoxicity of the pregnanone from this genus.

## Experimental

### General

Melting points were obtained on Beijing Taike X-5 stage apparatus uncorrected. The NMR spectra were recorded on Bruker AV-400 spectrometer, using TMS as an internal standard. The FAB-MS spectra were measured with a VG Autospec-3000 mass spectrometer, and the HRESI-MS spectra were measured with an API QSTAR Pulsar mass spectrometer. The IR spectra were obtained on a Nicolet 380 FT-IR instrument, as KBr pellets. The UV spectra were measured on a Beckman DU800 spectrometer. Optical rotation was recorded using Rudolph Autopol III polarimeter (U.S.A). Column chromatography was performed with silica gel (Marine Chemical Industry Factory, Qingdao, P.R. China), and Macroporous resin D101 (Shandong Lukang Pharmaceutical Co., Ltd.). TLC was preformed with silica gel GF254 (Marine Chemical Industry Factory, Qingdao, China), and developed by spraying with 10% H_2_SO_4_ followed by heating.

### Plant material

The roots of *Calotropis gigantea* used in this research were collected from Eman Village of Danzhou County, Hainan Province, P. R. China, in December 2006, and authenticated by Prof. Zhu-Nian Wang of the Institute of Tropical Crops Genetic Resources, Chinese Academy of Tropical Agricultural Sciences. The voucher specimen (No 20061201) was deposited at the Institute of Tropical Crops Genetic Resources, Chinese Academy of Tropical Agricultural Sciences.

### Extraction and isolation

The roots (26.7 kg) of *Calotropis gigantea* were extracted three times with 95% ethanol at room temperature. Following filtration, the combined ethanol extract was evaporated to dryness under reduced pressure to give a crude extract. The crude ethanol extract was suspended in water (6.0 L) and successively partitioned with petroleum ether to give Petro-soluble fraction (236.1 g) and an aqueous residue. Then the aqueous residue was concentrated and applied to a D-101 resin column, eluting with H_2_O and MeOH, successively, the MeOH eluent was collected and evaporated under reduced pressure to afford the “MeOH fraction” (yield 256.1 g). The MeOH fraction was subjected to vacuum liquid chromatography (VLC) over silica gel, eluting with gradient elution CHCl_3_-MeOH (100:0, 50:1, 25:1, 10:1, 5:1, 2:1, MeOH) to afford seven fractions (Fr.1−Fr.7). Fr.1 (56.3 g) was subjected to further column chromatography over silica gel, with petroleum ether-acetone (6:4) as eluent, to afford compound **1** (32 mg). Fr.4 (20.3 g) was subjected to column chromatography over silica gel, eluting with gradient elution CHCl_3_-MeOH to afford **2** (35 mg).

*Calotropone* (**1**): Yellow gum, [α]
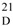
−89.7° (*c* 0.26, MeOH); HR-ESI-MS: *m/z* [M+Na]^+^ 491.2409( calcd. For C_28_H_36_O_6_Na, 491.2404); IRν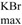 (cm^-1^): 3431, 2918, 2849, 1712, 1629, 1463, 1275, 1110; UVλ_max_ nm (CHCl_3_): 241, 267, 284; ^1^H-NMR (400 MHz, CDCl_3_), ^13^C-NMR (100 MHz, CDCl_3_ ): Table 1.

*Gofruside* (**2**) [15,16]: C_2__9_H_40_O_9_, Colorless needles; m.p. 165 − 167 °C; IR (KBr) λ_max_ (cm^-1^): 3437, 2937, 1738; FAB-MS (neg.) *m/z* 535 [M−H]^−^; ^1^H-NMR (400Hz, CD_3_OD): *δ* 5.88 (1H, *br*s, H-22), 5.02, 4.89 (each 1H, d, *J* = 18.3 Hz, H-21), 4.70 (1H, d, *J* = 7.9 Hz, H-1'), 3.69 (1H, m, H-3), 3.65 (1H, m, H-5'), 3.29 (1H, brs, H-3'), 3.23 (1H, dd, *J* = 2.5, 7.7 Hz, H-2'), 3.14 (1H, dd, *J* = 1.8, 9.4 Hz, H-4'), 1.20 (3H, d, *J* = 6.0 Hz, H-6'), 0.91 (3H, s, H-18); ^13^C-NMR (100 MHz, CD_3_OD): *δ* 32.1 (C-1), 30.2 (C-2), 78.7 (C-3), 37.0 (C-4), 45.1 (C-5), 30.0 (C-6), 28.6 (C-7), 43.8 (C-8), 51.9 (C-9), 52.8 (C-10), 22.9 (C-11), 40.6 (C-12), 50.9 (C-13), 86.1 (C-14), 32.8 (C-15), 27.9 (C-16), 52.0 (C-17), 16.3 (C-18), 210.4 (C-19), 178.3 (C-20), 75.3 (C-21), 117.9 (C-22), 177.2 (C-23), 99.9 (C-1'), 72.4 (C-2'), 72.9 (C-3'), 74.3 (C-4'), 70.5 (C-5'), 18.2 (C-6').

### Cytotoxicity bioassay

Compounds **1** and **2** were examined for their cytotoxic activity against chronic myelogenous leukemia K562 and human gastric cancer SGC-7901 cell lines. Cancer cells were incubated for 3 days at 37 °C in the presence of various concentrations of compounds from DMSO-diluted stock solutions. The growth inhibitory property was determined using 3-(4,5-dimethylthiazol-2-yl)-2,5-diphenyltetra-zolium bromide (MTT) assay as described by Mosmann [14].
