# Correction: Wang, Z.-N., *et al*. A New Cytotoxic Pregnanone from *Calotropis gigantean*. *Molecules* 2008, *13*, 3033-3039

**DOI:** 10.3390/molecules14010412

**Published:** 2009-01-15

**Authors:** Zhu-Nian Wang, Mao-Yuan Wang, Wen-Li Mei, Zhuang Han, Hao-Fu Dai

**Affiliations:** 1 Institute of Crops Genetic Resources, Chinese Academy of Tropical Agricultural Sciences, Danzhou, 571737, P. R. China; E-mails: wmy81@163.com (M-Y. W.); wangzhunian@yahoo.com.cn (Z-N. W.); 2 Institute of Tropical Bioscience and Biotechnology, Chinese Academy of Tropical Agricultural Sciences, Haikou, 571101, P. R. China; E-mails: hanzone@yahoo.cn (Z. H.); meiwenli@yahoo.com.cn (W-L. M.)

We wish to report the following errors in our paper recently published in Molecules [1]: on page 3035, the correct name of compound **1** should be 12-*O*-benzoyl-3,14,17-trihydroxypregn-5-en-20-one. Then, in Figure 1 on page 3034, compound **2** should appear as saturated at C5-C6 with a 5-α H. On page 3037, the molecular formula of compound **2** should be C_29_H_42_O_9_, its m.p. should be 265 − 267 °C, and the FAB-MS (neg.) of compound **2** should be *m/z* 533 [M−H]^−^. Figure 1 below shows the revised structures of compounds **1** and **2**. The authors apologize for any inconvenience to the readership.

**Figure 1:**
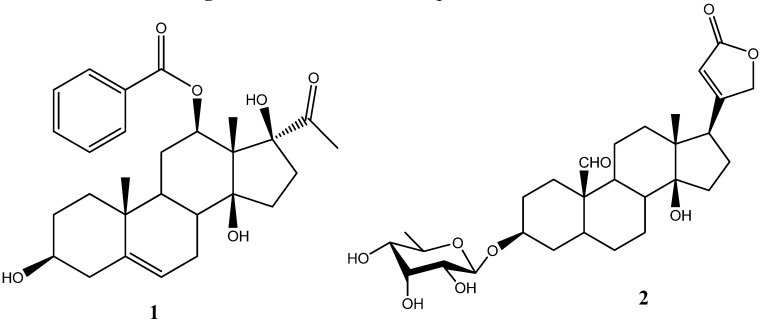
Structures of Compounds **1** and **2**.
